# Association of Antiosteoporotic Medication Bisphosphonates and Denosumab with Primary Breast Cancer: An Electronic Health Record Cohort Study

**DOI:** 10.1089/whr.2020.0120

**Published:** 2021-08-16

**Authors:** Alexander Stanoyevitch, Lei Zhang, Javier Sanz, Robert W. Follett, Douglas S. Bell

**Affiliations:** ^1^ Department of Mathematics, California State University-Dominguez Hills, Carson, California, USA.; ^2^ Pathology Associates of Anaheim, Anaheim Regional Medical Center, Anaheim, California, USA.; ^3^ University of California at Los Angeles, Clinical and Translational Science Institute, Los Angeles, California, USA.

**Keywords:** bisphosphonates, breast cancer, denosumab, osteoporosis, statins

## Abstract

***Background:*** The risks of osteoporosis and breast cancer are increasing in elderly women. Bisphosphonates and denosumab are recommended for treatment of osteoporosis. They have different and overlapping pharmacodynamics and previous studies have shown conflicting results regarding their risk association with breast cancer. We intend to further look into this issue through a comparative study.

***Methods:*** Electronic health records of 91,626 women older than 50 years with no previous history of malignancy and no nonbreast cancer during follow-up were retrieved from southern California and retrospectively analyzed using univariate, bivariate, and log-rank tests. Medication use, breast cancer risk, and associated demographic and clinical history were assessed.

***Results:*** Over an average of 3.6 years follow-up, the breast cancer relative risks (RRs) counted after 365 days of latency are 1.12 (95% confidence interval [CI]: 0.64–1.97) for denosumab ever users and 0.37 (95% CI: 0.21–0.66) for bisphosphonates ever users, when covariates are comparable. The significant difference is supported by the Log-rank test (*p* = 0.0004). Excluding statins coprescribers, the breast cancer RR is 1.31 (0.71, 2.43) in denosumab group and 0.26 (0.11, 0.62) in bisphosphonates group. There is a reduced RR in statins ever users (0.47, 95% CI: 0.38–0.58), and the breast cancer risk difference is not significant between concomitant denosumab/statins and bisphosphonates/statins ever users with RR 0.65 (0.16, 2.58) versus 0.55 (0.26, 1.16), *p* = 0.692.

***Conclusions:*** Our data support an association of lower breast cancer risk with bisphosphonates use in elderly women. We did not observe a lower breast cancer risk in denosumab group; however, our data revealed a potential lower breast cancer risk in denosumab users with concurrent statins use and this requires further study.

## Introduction

Both osteoporotic fracture and cancer can be a devastating personal and social economic burden, and the risks for both are increasing when the modern life expectancy is growing over the age of 80 years. Seventy-one percent of osteoporotic fractures occur in women, while breast cancer is the most common tumor all over the world.^1,2^ The effects of antiosteoporosis medication on breast cancer are a debate and a concern.

Currently, bisphosphonates (Alendronate, risedronate, and zoledronic acid) and denosumab are recommended by United States Preventive Services Task Force (USPSTF) to treat osteoporosis in postmenopausal women younger or older than 65 years.^1^

The antiosteoporotic effects of bisphosphonates and denosumab are different: denosumab is more effective and faster in improving bone mass density; but effects of bisphosphonates continue for years after drug discontinuation because they are imbedded in the bone, while denosumab discontinuation fully and rapidly reverse its effects on bone markers and bone mineral density. Denosumab is contraindicated in severe infection, but is preferred in patients with renal failure.^3–5^

Epidemiological studies have also shown that bisphosphonates are associated with variable nonadverse, for example, protective or no related risk toward female breast cancer.^6–15^ A recent study suggests a potential protective effect of denosumab ever use on breast cancer risk in a cohort of older women previously treated with bisphosphonates.^16^ It is not clear whether this protective effect is due to lingering effects of previous bisphosphonates use. Denosumab is active in body for only 6 months compared to years of lingering effects from bisphosphonates. This may lead to a speculation that denosumab has no effect on breast cancer in women who took and discontinued it. However, drug side effects may not occur until long time after stopping the medication and cancer can be related to radiation exposure events, which have lasted only minutes. A comparative study of denosumab and bisphosphonates might be helpful addressing this question. An initial epidemiological study based on the Nurses' Health Study (NHS) cohort did not show protective effects of statins on breast cancer, when antiosteoporosis therapy was not considered in either medication or control groups.^17^

Cancer risk reduction using pharmacological means is an attractive modern preventive approach that supplements the classical behavioral prevention recommendations. Studying commonly used drugs such as bisphosphonates and statins as candidate cancer chemopreventive agents has the advantage of usually having a low-risk profile and is associated with much clinical experience.

The osteoporosis risk increases after menopause, which is on average by age of 51, and this is also the early starting age antiosteoporosis medications are provided. We set the start of our observation time as age 50 and older.

The potential different effects of bisphosphonates and denosumab on breast cancer have not been compared yet. How possibly the drug interaction of antiosteoporosis with statins could further modify breast cancer risk is unknown. Knowledge of these may help decisions on individualized medication best beneficial to patients. We aim to investigate those questions using a cohort, including females 50 years of age or older.

## Materials and Methods

### Population

This study was approved by the Research Ethics Board of University of California at Los Angeles (UCLA), IRB#16-000581. Inclusion criteria: female and age 50 or older at their first visit, with at least two ambulatory encounters in 1 year. Exclusion criteria: previous diagnosis of cancer in the first encounter and cancer diagnosis other than breast cancer during follow-up.

### Data retrieval

The clinical data were retrieved from electronic health record (EHR) Epic. A 3.6-year duration of chronological clinical information was extracted from the Clarity data base, which has been daily transferred from the Epic application (Chronicles). The requested data output for this project is in nine CSV (comma-separated values) format files, including parameters of ICD, SNOMED diagnoses, laboratories, medications, family history, allergies, vital signs, and demographic information. The medications include prescriptions linked to pharmacy fill-up or in-house administration. All HIPAA (Health Insurance Portability and Accountability Act) identifiers have been stripped from the data sets. These data sets were linked using unique encoded identifiers.

### Analytics

#### Study groups

In addition to denosumab and bisphosphonates, we also examined possible interactions with two other popular drug classes: hormones and statins. The patients were separated into two mutually exclusive groups, medication group and control group:

#### Medication group

It includes (1) denosumab, (2) bisphosphonates (Alendronate, Risedronate, Zoledronic, Pamidronate, and Ibandronate), (3) statins (Simvastatin, Atorvastatin, Rosuvastatin, Fluvastatin, Pitavastatin, Lovastatin, and Pravastatin), and (4) hormones (used by women to reduce menopausal symptoms, including patch, tablets or vaginal ring of estradiol, estrogen, and Norethindrone Acetate-Ethinyl Estradiol).

#### Control group definition

Hospital visitors who have never been prescribed bisphosphonates, denosumab, statins, or hormones.

#### Breast cancer identification

ICD-9 (174.9, V10.3) and ICD-10 (C50, Z85.3) codes, which are authoritative tools for disease identification, besides their association with claims and reimbursement, are used to identify breast cancer patients (see Supplementary Fig. S1 for initial validation study).

#### Breast cancer patients definitions

(1) Patients who were diagnosed with breast cancer 365 days or later after they were first prescribed any of these four medication groups; (2) patients in the control group who were diagnosed with breast cancer at least 365 days after the first encounter. The following situations are excluded from counting of breast cancer cases to focus on primary breast cancer study, and to exclude situations when denosumab/bisphosphonates are used to treat bone metastatic tumor or myeloma: (1) patients exposed to denosumab or bisphosphonates who have cancer diagnoses other than breast type; (2) patients with breast cancer diagnosis before denosumab or bisphosphonates administration.

#### Covariates

We examined controlling our comparisons against the following relevant covariates: age, body mass index (BMI), blood pressure (BP), hyperlipidemia, diabetes status, breast cancer family history, and alcohol ever use. Those parameters are extracted from either ICD-diagnosis codes or laboratory measurements or encounter documentation.

### Statistical analysis

All data analyses and application of inclusion and exclusion criteria were performed using the R statistical software package (with R Studio).

The univariate and bivariate analyses include a Kaplan-Meier plot and its associated log-rank test, relative risks (RRs), confidence intervals (CIs), and Fisher's exact test, and *p* values (one or two-tail, significance level: *p* < 0.01) were provided whenever feasible.

For each patient in the medication group, we computed the last date of any of the four drugs that they took and added that number to 365 to determine the threshold after which a breast cancer diagnosis gets counted.

## Results

### Formation of study cohort

**FIG. 1.**
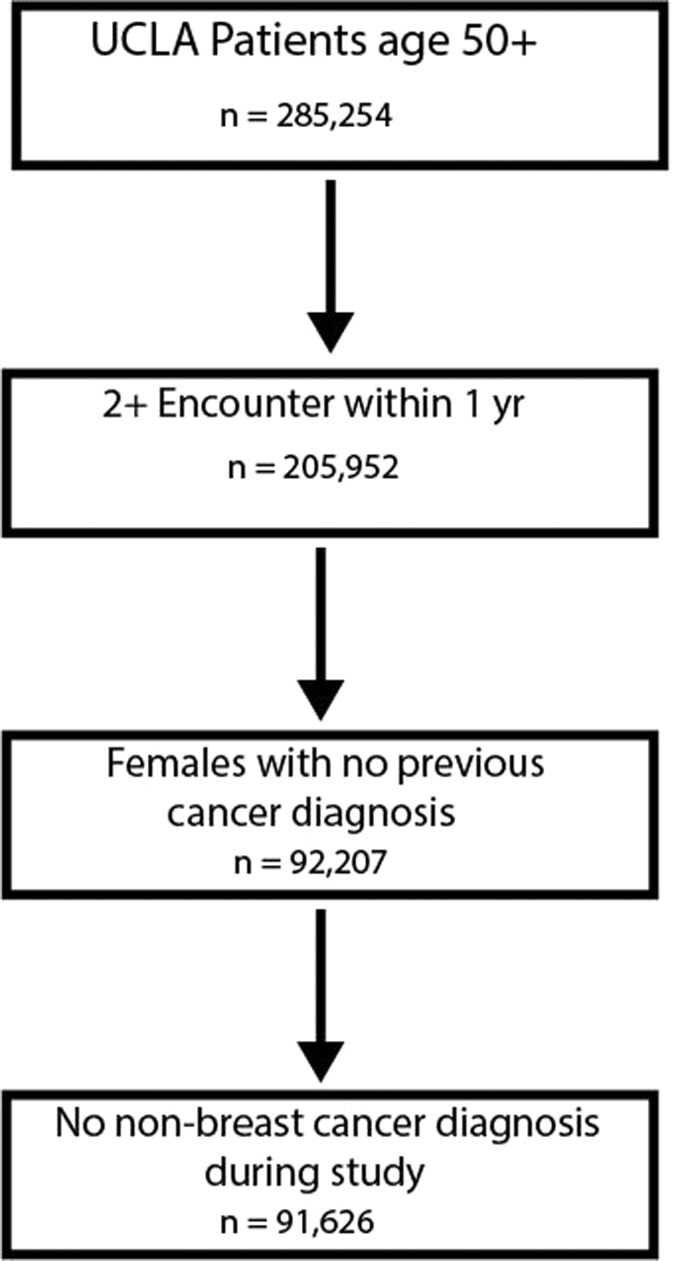
Diagram of cohort formation. UCLA, University of California at Los Angeles.

The UCLA health system has around 180 primary and specialty care practices in southern California. There are 285,254 patients who are aged 50 years or older at their first visit on or after date of January 01, 2012. The visit types include all encounters documented in the Epic EHR, including ambulatory (outpatient clinics, physician offices, same day/ambulatory surgery centers, urgent care facilities, and other same-day ambulatory hospital encounters), emergency, emergency to inpatient, and inpatient, etc. 205,952 patients who are older than 50, with at least two ambulatory encounters in 1 year from January 1st, 2012 to July 1st, 2016 in UCLA electronic health system were first sorted out. After excluding male patients and patients who have had previous diagnosis of cancer in first encounter and cancer diagnosis other than breast cancer during follow-up, our cohort includes 92,207 female patients. Excluding patients with cancers other than breast, a total of 91,626 patients enter final analysis (Fig. 1).

### Differential breast cancer risk between denosumab and bisphosphonates group

The duration of denosumab and bisphosphonates use in our cohort is mostly around 1–2 years. The number of patients taking the medications and prescription dosage are summarized in Supplementary Table S1.

**FIG. 2.**
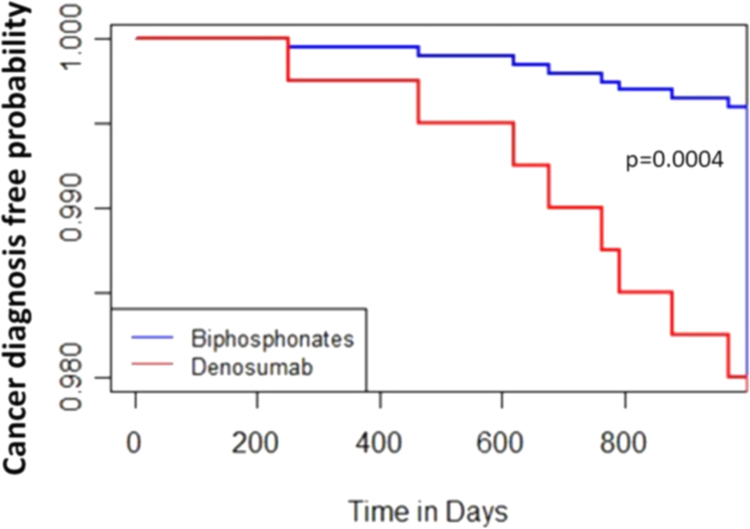
Breast cancer free diagnosis in bisphosphonates and denosumab users, Kaplan-Meier plot and log-rank test.

Excluding the first-year latency, the absolute breast cancer risk in denosumab ever use group is 1.54% (12/778), compared to 0.52% (12/2326) in bisphosphonates, with RR of 1.12 (95% CI: 0.64–1.97) versus 0.37 (95% CI: 0.21–0.66). The accumulative risk is statistically significant (*p* = 0.0085) [Table 1]. The breast cancer distribution in follow-up time of 3 years as shown by Kaplan-Meier plot and log-rank test is also significant between the two groups (*p* = 0.0004) (Fig. 2). The covariates between the two groups are comparable (Table 2, a concise summary in Supplementary Table S2).

**Table 1.** Comparison of Breast Cancer Risks Among Different Medication Groups

	Breast cancer risk & 95% CI	Breast cancer RR^a^ (95% CI)	p-(RR of denosumab ≠ bisphosphonates)
Medication ever users			
Denosumab	1.54% (12/778) 0.68%, 2.41%	1.12 (0.64–1.97)	0.0085
Bisphosphonates	0.52% (12/2326) 0.22%, 0.81%	0.37 (0.21–0.66)	
Statins	0.65% (99/15,287) 0.52%, 0.78%	0.47 (0.38–0.58)	
Hormone (for postmenopausal symptoms)	0.26% (20/7631) 0.15%, 0.38%	0.19 (0.12–0.30)	
Comedication ever users			
Denosumab+statins	0.89% (2/224) 0.24%, 3.90%	0.65 (0.16–2.58)	0.6920
Bisphosphonates+statins	0.76% (7/919) 0.37%, 1.56%	0.55 (0.26–1.16)	
Single medication ever users			
Denosumab	1.81% (10/554) 0.99%, 3.30%	1.31 (0.71–2.43)	0.0023
Bisphosphonates	0.36% (5/1407) 0.15%, 0.84%	0.26 (0.11–0.62)	
Control	1.38% (1032/74,867) 1.30%, 1.47%	1	

^a^ RRs are comparisons to control population.

CI, confidence interval; RR, relative risk.

**Table 2.** Stratified Breast Cancer Risk in Different Medication Groups

	Denosumab N = 778	Bisphosphonates N = 2326	Statins N = 15,287	Hormone N = 7631	Control N = 74,867
Age of first encounter (first quarter, median, mean, third quarter)	62, 68, 69.47, 78	61, 67, 68.59, 76	60, 67, 68.23, 75	55, 60, 61.9, 67	56, 63, 64.47, 71
Family history of breast cancer (%, *n*)	15% (119)	12% (273)	9% (1441)	14% (1052)	7% (5187)
Alcohol ever use (%, *n*)					
Yes	33% (254)	29% (666)	31% (4708)	45% (3446)	29% (21,815)
No	46% (356)	48% (1128)	44% (6659)	31% (2372)	33% (24,469)
Not asked or missing data	21% (168)	23% (532)	25% (3920)	24% (1813)	38% (28,583)
BMI					
Mean	24.99	24.91	27.88	25.15	26.54
>30	15%	14%	14%	15%	24%
Breast cancer absolute risk	1.00%	0.00%	0.68%	0.33%	2.34%
<25	51%	59%	36%	57%	47%
Breast cancer absolute risk	1.50%	1.13%	0.71%	0.20%	2.35%
RR (95% CI)^a^	0.638 (0.28–1.44)	0.489 (0.27–0.85)	0.301 (0.21–0.44)	0.088 (0.04–0.19)	1.000
BP					
Mean (systolic/diastolic)	127/73	128/72	130/73	125/73	128/74
Hypertension (>140/>90) (%)	26%	29%	33%	21%	31%
Breast cancer absolute risk	2.87%	0.97%	0.84%	0.47%	2.87%
Normal (<120/<80) (%)	42%	42%	36%	51%	43%
Breast cancer absolute risk	1.92%	0.93%	0.77%	0.27%	1.83%
RR (95% CI)^a^	1.052 (0.47–2.38)	0.509 (0.24–1.08)	0.420 (0.29–0.61)	0.145 (0.07–0.29)	1.000
Diabetes					
Diabetes diagnosis (%)	10%	9%	15%	6%	15%
Breast cancer absolute risk	1.92%	0%	0.92%	0.47%	1.35%
No-diabetic diagnosis	90%	91%	85%	94%	85%
Breast cancer absolute risk	1.57%	0.85%	0.60%	0.25%	1.38%
RR (95% CI)^a^	1.136 (0.62–2.07)	0.618 (0.39–0.99)	0.435 (0.35–0.55)	0.181 (0.11–0.29)	1.000
Lipid panel					
Hyperlipidemia documented (%)	23%	23%	38%^b^	18%	5%^c^
Breast cancer absolute risk	1.11%	1.90%	0.90%	0.22%	2.51%
No hyperlipidemia documented					
breast cancer absolute risk	1.68%	0.44%	0.49%	0.27%	1.32%
RR (95% CI)^a^	1.268 (0.68–2.38)	0.337 (0.17–0.68)	0.370 (0.17–0.68)	0.205 (0.13–0.33)	1.000

^a^ RRs are for strata with normal value, compared to control population.

^b^ Statins are not only indicated for hyperlipidemia situation, but also recommended to optimize lipid levels in diabetes even if those patients may not qualify for the diagnosis of hyperlipidemia.

^c^ The low number is largely due to removal of statin treatment group from the control.

BMI, body mass index; BP, blood pressure; RR, relative risk.

Among denosumab and bisphosphonates ever users, only 84 (84/3020 = 2.8%) patients have taken both medications during follow-up. Moreover, excluding statins coprescribers, the breast cancer RR is still significantly different between denosumab group and bisphosphonates group [1.31 (0.71, 2.43) vs. 0.26 (0.11, 0.62), *p* = 0.0023] [Table 1].

### Paradoxically lower breast cancer risk in hyperlipidemic stratification in denosumab group

We next examine whether this risk difference between denosumab and bisphosphonates continues to hold in the stratifications of risk factors reflexing general health and physical activity.

Of note, in the medication groups of denosumab, bisphosphonates, statins, and hormones, 3218 women out of 26,022 have taken more than one type of the four medications, accounting for 12% (3218/26,022) of the aforementioned medication prescribers. The control group is hospital or office visitors (*n* = 74,867) who have never been prescribed bisphosphonates, denosumab, statins, or hormones.

Our data analyses showed that denosumab and control groups have similar breast cancer risk (1.54% vs. 1.38%, *p* = 0.6965); the breast cancer risks in bisphosphonates (0.52%), statins (0.65%), and hormone (0.26%) groups are significantly lower than control (*p* = 0.0004, <0.0002, <0.0002) [Table 1]. The differences of breast cancer risk hold constant in stratifications of BMI, BP, and diabetes status [Table 2]. In blood lipid level stratification, however, the breast cancer risk is paradoxically lower in denosumab group (1.11%, 2/181) compared to that in bisphosphonates group (1.90%, 10/526) in hyperlipidemic patients, although the risks in normal lipid group are the opposite (higher in denosumab 1.68%, lower in bisphosphonates 0.44%) [Table 2]. This has raised a concern that lipid lowering medications such as statins may confound breast cancer risk in denosumab users.

Joint use of statins is associated with lower breast cancer risk in the denosumab group but not in the bisphosphonates group

The concurrent statins use is 29% (224/778) in denosumab groups and 40% (919/2326) in bisphosphonates group (*p* < 0.001%). When we looked at the breast cancer risk in joint medication users, we found that joint denosumab and statin use showed a lower breast cancer RR compared to denosumab ever use (0.65, 95% CI: 0.16–2.58 vs. 1.12, 95% CI: 0.64–1.97), but the association is not statistically significant. The wide 95% CI (0.16–2.58) observed in joint denosumab and statin users is due to few (*n* = 2) cancer cases observed. In contrast, joint use of bisphosphonates and statins show a breast cancer RR slightly higher than single medication ever use, but not statistically significant (0.55, 95% CI: 0.26–1.16 vs. 0.55, 95% CI: 0.26–1.16) [Table 1].

### Other findings

The hormone group in this cohort is characterized by lowest proportions of hyperlipidemia, diabetes, hypertension, and highest proportion of lean body figure (BMI less than 25) compared to other groups. This is associated with lowest breast cancer risk among all the groups despite that this group has a higher incidence of breast cancer family history.

Of note, the proportion of hyperlipidemia is low in the control. This is largely due to removal of statin treatment group from the control. In addition, only 38% of patients in statins group have a diagnosis of hyperlipidemia, this is because statins are not only indicated for hyperlipidemia situation, but also recommended to optimize lipid levels in diabetes even if those patients may not qualify for the diagnosis of hyperlipidemia.^18^

### Missing data

The numbers of missing data vary among categories. Alcohol ever use category has the highest frequency of missing data (up to 25%). Nonetheless, there is no significant difference of missing data among the four medication groups and control group. Missing data were excluded from analysis.

## Discussion

### Rationale of medication grouping and covariates selections using data from EHRs

Both denosumab and bisphosphonates are approved by FDA to treat bone metastatic solid tumors or hematopoietic tumor myeloma involving bone. Those clinical scenarios are excluded from this study by excluding any patients with malignant diagnosis in first encounter, and any cancer diagnosis other than breast carcinoma during follow-up, and applying at least 365-day waiting time in medication groups for breast cancer case counting (any breast cancer cases diagnosed 365 days before medication or within 365 days after first encounter were dropped off from analysis).

Denosumab is administrated subcutaneously every 6 months. Bisphosphonates are administered at variable interval of daily, weekly, quarterly, or yearly regime. The half life of bisphosphonates is up to years and denosumab still has a detectable serum level 9 months or later. The optimal duration of antiosteoporosis treatment has not been established. On the contrary, the reported timing of bisphosphonates use associated with breast cancer reduction has a wide variation: some found that the effect existed only after at least 1 year^7,9^; others said that deduction was not duration dependent^6^; some indicated that it was present only among women with <2 years of use^12^; others suggested that it was more marked with increasing duration of use.^8^ The only denosumab and breast cancer association report studied women who filled a first prescription for denosumab (denosumab ever use).^16^ In our cohort, the average duration for both denosumab and bisphosphonates use is around 1–2 years and the dosage is shown in Supplementary Table S1. It is uncertain whether various dosages are responsible to the confusing results regarding the estimated effect of bisphosphonates according to the timing of use. Because of the lingering effect of bisphosphonates and low-frequency standard administration regime for denosumab, we classify the antiosteoporosis therapy as two category dichotomous binary data, for example, with or without treatment.

Hormone treatment is required for menopausal symptom control in some patients due to decreased endogenous estrogen level. The hormone treatment may indicate both decreased risk for breast cancer (lower endogenous hormone level) and increased risk (supplemental hormone). Hormone users are a highly selected group. We therefore separate this group from our control.

Well-accepted breast cancer risks include age, family history, imbalanced estrogen level, adiposity (BMI), and alcohol ever use.^2,19^ Smoking might have an initiation role in breast cancer, although no causal relationship is suggested.^2^ Moreover, diabetes status, which has not been mentioned as a risk factor for breast cancer in World Health Organization (WHO) Classification of Tumors of the Breast, has been reported to be associated with breast cancer.^20,21^ We included those in our covariate analysis. We compared parameters of BP and lipidemia, which are related to general health and physical activity. The covariate analysis showed that there is no significant biomedical difference between the denosumab and bisphosphonates ever use groups.

### Comparison of our results with other studies

There were nine large studies evaluating bisphosphonates use and primary breast cancer risk in different geographic area and in various populations before this study. Three case control studies^6–8^ and two cohort studies^9,11^ suggest protective role of bisphosphonates toward breast cancer, and the other four studies^10,12–14^ did not prove significant protective effect, although no adverse effect is identified. The pooled results of those data^15^ showed that bisphosphonates were associated with 12% decrease risk of primary breast cancer (RR: 0.88; 95% CI: 0.83–0.94). Our study favors an association of significantly decreased breast cancer risk with bisphosphonates use.

The most recent large French cohort study^10^ observed a decrease in breast cancer risk associated with bisphosphonates use restricted to the year after treatment initiation (RR: 0.56; 95% CI: 0.36–0.87). This is close to our finding which also showed breast cancer RR 0.37 (95% CI: 0.21–0.66) in bisphosphonates ever use patients. Per meta-analysis,^15^ the observed association of primary breast cancer risk with long-term use (≥1 year) of bisphosphonates seemed to be more robust and stronger than that of short-term use (<1 year) (RR: 0.75; 95% CI: 0.66–0.84; and 0.90; 95% CI: 0.84–0.97; respectively). However, the only randomized control trials showed that 3–4 years of bisphosphonate treatment did not decrease the risk of invasive breast carcinoma in postmenopausal women.^14^ It is noted that this randomized control study was not initially designed to study breast cancer outcome. Future large randomized control studies are required to verify this concern.

Different from bisphosphonates whose cancer association has been studied for a decade, there is only one article recently published addressing the relationship between denosumab and breast cancer risk. This first case-control study showed that in a cohort of older women previously treated with bisphosphonates, denosumab use was associated with a 13% decreased breast cancer risk (Hazard Ratio = 0.87; 95% CI: 0.76–1.00).^16^ There was no relationship between increasing number of denosumab doses and breast cancer risk (*p*-trend = 0.15).^16^ Our cohort study, which has a similar length of follow-up to the former study but with a bisphosphonates and denosumab comedication rate of 2.8% (84/3020 = 2.8%), did not come to the same conclusion. We showed that breast cancer risk in denosumab users is not significantly different from the control, although our study demonstrated association of lower breast cancer risk with bisphosphonates.

The initial epidemiological study on statins and breast cancer association is from the NHS cohort.^17^ It showed no associated risk of breast invasive carcinoma in statins users, but the comedication analysis did not include bisphosphonates use. All the cohort and case-control studies, which focused on the relationship of bisphosphonates and breast cancer, did not separate statins use from control group either.^6–14^ In our study, statins are associated with similar breast cancer protective effect as bisphosphonates. This is in consistence with published preclinical research.^22–24^ It is possible that the comparable breast cancer protective effect of bisphosphonates or statins might be masked when the control group has comedication of either of these two drugs.

We also showed that comedication of denosumab and statins is associated with lower cancer risk compared to denosumab ever use group, although the cancer cases (events) are less than 5, and statistical significance is hard to evaluate [Table 1]. Plans are underway for our acquiring even larger medical records data sets to further investigate such concepts.

This healthier biomedical status in the hormone group (lowest proportions of hyperlipidemia, diabetes, hypertension, and highest proportion of lean body figure) may explain the lowest breast cancer risk among all the groups, despite this group has a higher incidence of breast cancer family history. The absence of increased breast cancer risk may also be related to low dose and formulation of supplementary hormone in this group of patients. There is a great discrepancy on breast cancer risk and postmenopausal hormone use.^2^ In a contemporary observational cohort study, more than 100,000 women aged 50 to 71 were followed prospectively for 15 years. It showed that long-term hormonal contraceptive use reduced ovarian and endometrial cancer risks by 40% and 34%, respectively, with no increase in breast cancer risk regardless of family history.^19^

### Preclinical studies on effects of bisphosphonates, denosumab, and statins toward breast cancer risk

Bisphosphonates and denosumab are both antiresorption drugs inhibiting the osteoclasts activity, but with different binding sites in the bone. Bisphosphonates bind to bone mineral matrix hydroxyapatite at the surface of bone and especially within the resorption lacunae, occupying the site of resorption performed by activated osteoclasts, where they could be internalized by the active osteoclasts and inhibit the intracellular mevalonate pathway, leading to impaired function and apoptosis of osteoclasts. Denosumab is a newer monoclonal antibody first approved by FDA for treatment of postmenopausal osteoporosis in June 2010. It suppresses bone resorption by binding to receptor activator of nuclear factor kappa-B ligand (RANKL), preventing it from binding to its receptor on cell surfaces of not only osteoclasts but also osteoclasts precursors and decreasing osteoclast formation, activity, and survival.^3^

The pharmaceutical effects of bisphosphonates are mediated by estrogen related receptor α (ERRα).^25^ ERRα plays roles in osteoporosis and breast cancer development. ERRα transcriptional activity is enhanced by cholesterol and suppressed by statins and bisphosphonates.^22^ Meanwhile, the epigenetic impacts of statin and bisphosphonates on DNA methylation, histone deacetylation, and microRNAs occurring in normal cells could be both cancer preventing and promoting.^23^ RANKL/RANK/OPG system (receptor activator of nuclear factor/RANK ligand/osteoprotegerin) is not only critical for the regulation of osteoclast differentiation/activation and calcium release from the skeleton, but also can be regarded as a major downstream mediator of progesterone-driven mammary epithelial cells proliferation, potentially contributing to breast cancer initiation and progression.^24^ The regulatory network is summarized in Supplementary Figure S2.

The preclinical studies suggest possible protective role of bisphosphonates on breast cancer. The preclinical studies also suggest a potential synergistic effect between denosumab and statins on breast cancer reduction as well.

### Limitation

One might speculate that the antiosteoporosis medication group might have lower estrogen level than control. One of the factors leading to osteoporosis is plummet of estrogen at age 50 years and older. The estrogen level is mostly affected by age and positively related to BMI or obesity. Those two factors show no significant difference between the antiosteoporosis medication and control groups in our study. These may partially solve the indication bias concern. Preclinical studies (as summarized in Supplementary Fig. S2) suggest some antiestrogen effects of those antiosteoporosis medications, and this could explain the breast cancer risk reduction observed in this cohort study. A randomized control trial is optimal; however, this might be prevented by logistical and financial challenges.

The electronic health system records individualized personal care, in compliance with standard patient care. It enables us to analyze potential drug interaction among antiosteoporosis drugs, statins, and exogenous hormones in the real world. Although EHR has a strength of medication prescription retrievability, it also carries limitation of efficient and reliable documentation of other clinical information. Age at menarche and breastfeeding history are missing in majority of our EHRs. T-score of Dual-energy X-ray absorptiometry is absent for majority patients. Osteoporosis risk assessment FRAX score is not uniformly used and documented in EHR.

It is natural to ask whether the breast cancer patients in each medication subgroup have different prognosis. Breast cancer prognosis is currently stratified into eight groups based on anatomic characters, including T (tumor), N (nodes), M (metastasis), and biological types, including estrogen/progesterone receptor, and HER2 (Human Epidermal Growth Factor Receptor 2) status according to American Joint Committee on Cancer (AJCC) 8th edition.^26^ Furthermore, categorization based on genome, RNA, or protein expression profiles are also applied.^26^ Such complex stratifications require large number of breast cancer cases for testing of prognosis difference across each subgroup. This question may be addressed in future large-scale studies.

## Conclusion

This is the first study to compare the risk of primary breast cancer between bisphosphonates and denosumab users head-to-head. The results, if supported by further studies could be potentially helpful for clinical decision when breast cancer is a concern and patients are treated for osteoporosis with or without comorbidity of hyperlipidemia.

Our data support an association of lower breast cancer risk with bisphosphonates use in elderly women. We did not observe a lower breast cancer risk in denosumab group; however, our data revealed a potential lower breast cancer risk in denosumab users with concurrent statins use and this requires further study.

## Availability of Data and Materials

The data of this study are available from the Clinical and Translational Science Institute, UCLA. HIPPA restrictions apply.

## Ethics Declarations

This study was approved by the Research Ethics Board of University of California at Los Angeles.

## Supplementary Material

Supplemental data

Supplemental data

Supplemental data

Supplemental data

## Abbreviations Used

95% CI: 95% confidence interval
AJCC: American Joint Committee on Cancer
BMI: body mass index
CSV: comma-separated values
EHR: electronic health record
ERRα: estrogen-related receptor α
FDA: Food and Drug Administration
HER2: Human Epidermal Growth Factor Receptor 2
HIPPA: Health Insurance Portability and Accountability Act
ICD: International Classification of Diseases
NHS: Nurses' Health Study
NM: tumor nodes metastasis
OPG: osteoprotegerin
RANK: receptor activator of nuclear factor kappa-B
RANKL: receptor activator of nuclear factor kappa-B ligand
RR: relative risk
SNOMED: Systematized Nomenclature of Human Medicine
UCLA: University of California at Los Angeles
WHO: World Health Organization
