# Supplementary material for: Association of Antiosteoporotic Medication Bisphosphonates and Denosumab with Primary Breast Cancer: An Electronic Health Record Cohort Study
Source: Womens Health Rep (New Rochelle). 2021 Aug 16;2(1):316–24. doi: 10.1089/whr.2020.0120 (PMC8409235; doi:10.1089/whr.2020.0120)
Supplement: Supplemental data [file Supp_FigS1.docx]

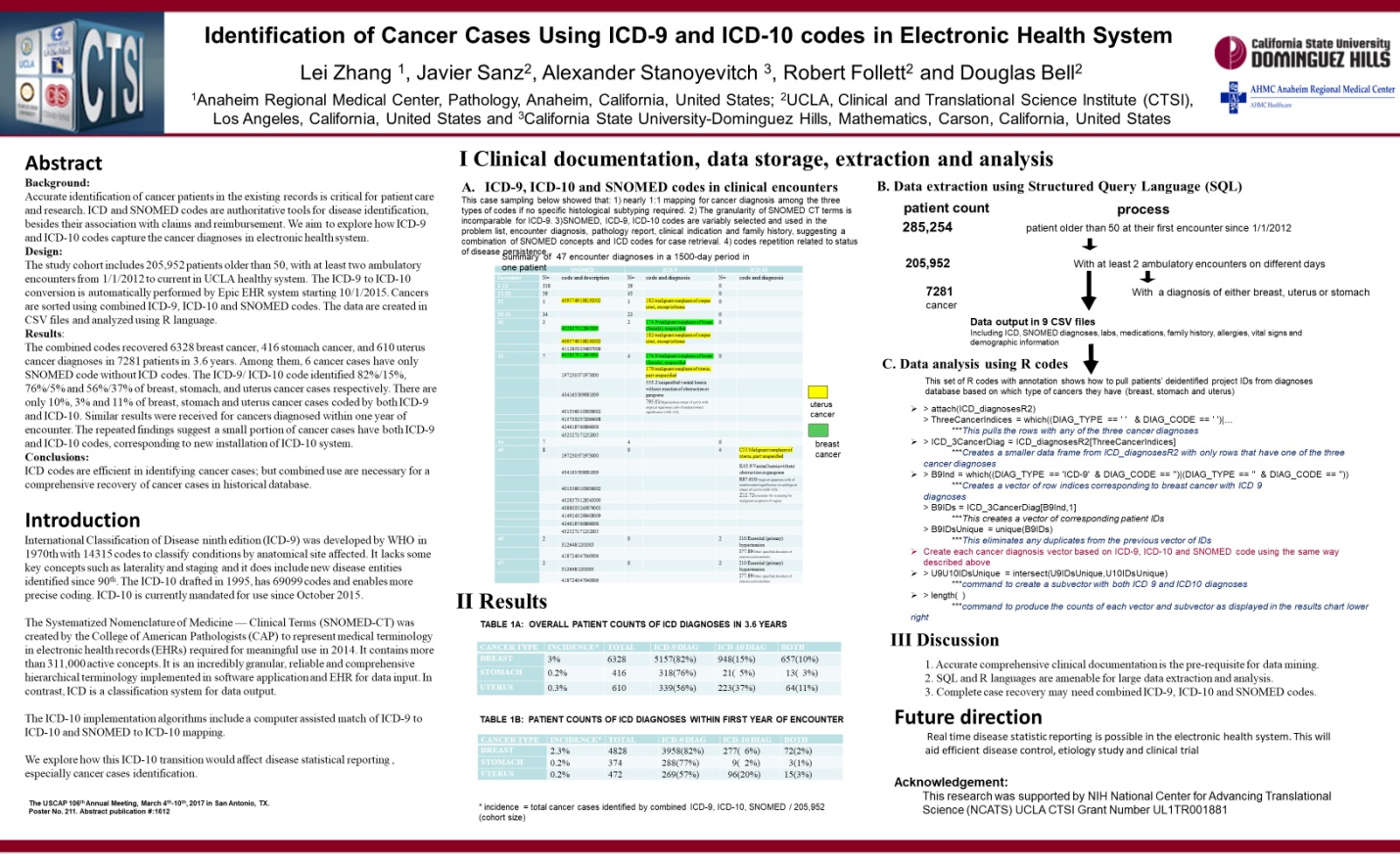


Supplementary Figure 1. Identification of cancer cases using ICD-9 and ICD-10 codes in electronic health system
