# Supplementary material for: Association of Antiosteoporotic Medication Bisphosphonates and Denosumab with Primary Breast Cancer: An Electronic Health Record Cohort Study
Source: Womens Health Rep (New Rochelle). 2021 Aug 16;2(1):316–24. doi: 10.1089/whr.2020.0120 (PMC8409235; doi:10.1089/whr.2020.0120)
Supplement: Supplemental data [file Supp_TableS1.docx]

Supplementary table 1. Accumulative exposure to bisphosphonates and denosumab

| Medication | No. of patients (n=) | Route of administration | All doses (mg) |
| --- | --- | --- | --- |
| Denosumab | 778 | Injection | 60, 120 |
| Alendronate | 2130 | Oral | 10, 35, 70 |
| Risedronate | 93 | Oral | 35, 150 |
| Zoledronate | 55 | Injection/intravenous | 4, 5 |
| Pamidronate | 45 | Intravenous | 90 |
| Ibandronate | 3 | Oral | 150 |
