# Supplementary material for: Association of Antiosteoporotic Medication Bisphosphonates and Denosumab with Primary Breast Cancer: An Electronic Health Record Cohort Study
Source: Womens Health Rep (New Rochelle). 2021 Aug 16;2(1):316–24. doi: 10.1089/whr.2020.0120 (PMC8409235; doi:10.1089/whr.2020.0120)
Supplement: Supplemental data [file Supp_TableS2.docx]

Supplementary table 2. Denosumab and bisphosphonates: breast cancer risk difference and comparable co-variates

|  | Denosumab  N=778 | Bisphosphonates  N=2326 | p= |
| --- | --- | --- | --- |
| Age of first encounter (first quarter, median, mean, 3rd quarter) | 62, 68, 69.47, 78 | 61, 67, 68.59, 76 |  |
| Family history of breast cancer (%, n=) | 15% (119) | 12% (273) |  |
| Hormone co-medication (%, n=) | 11% (87/778) | 12% (279/2326) |  |
| Alcohol ever use (%, n=) |  |  |  |
| Yes | 33% (254) | 29% (666) |  |
| No | 46% (356) | 48% (1128) |  |
| Not asked or missing data | 21% (168) | 23% (532) |  |
| Blood pressure (mean of systolic/diastolic) | 127/73 | 128/72 |  |
| Diabetes | 10% | 9% |  |
| Hyperlipidemia | 23% | 23% |  |
|  |  |  | >=0.01 |
|  |  |  |  |
| Breast cancer absolute risk | 1.542% (12/778) | 0.516%(12/2326) | 0.0046 |
